# Satisfaction with service delivery among HIV treatment clients enrolled in differentiated and conventional models of care in South Africa: a baseline survey

**DOI:** 10.1002/jia2.26233

**Published:** 2024-03-25

**Authors:** Idah Mokhele, Amy Huber, Sydney Rosen, Jeanette L. Kaiser, Nkgomeleng Lekodeba, Vinolia Ntjikelane, Cheryl Hendrickson, Nancy Scott, Sophie Pascoe

**Affiliations:** ^1^ Health Economics and Epidemiology Research Office Faculty of Health Sciences University of the Witwatersrand Johannesburg South Africa; ^2^ Department of Global Health Boston University School of Public Health Boston Massachusetts USA; ^3^ Department of Global Health, Amsterdam Institute for Global Health and Development Amsterdam University Medical Center, University of Amsterdam Amsterdam The Netherlands

**Keywords:** antiretroviral therapy, differentiated service delivery, HIV, healthcare quality, patient satisfaction, South Africa

## Abstract

**Introduction:**

Differentiated service delivery (DSD) models aim to increase the responsiveness of HIV treatment programmes to the individual needs of antiretroviral therapy (ART) clients to improve treatment outcomes and quality of life. Little is known about how DSD client experiences differ from conventional care.

**Methods:**

From May to November 2021, we interviewed adult (≥18) ART clients at 21 primary clinics in four districts of South Africa. Participants were enrolled consecutively at routine visits and stratified into four groups: conventional care‐not eligible for DSD (conventional‐not‐eligible); conventional care eligible for but not enrolled in DSD (conventional‐not‐enrolled); facility pickup point DSD model; and external pickup point DSD model. Satisfaction was assessed using questions with 5‐point Likert‐scale responses. Mean scores were categorized as not satisfied (score ≤3) or satisfied (>3). We used logistic regression to assess differences and report crude and adjusted odds ratios (aORs). Qualitative themes were identified through content analysis.

**Results:**

Eight hundred and sixty‐seven participants (70% female, median age 39) were surveyed: 24% facility pick‐up points; 27% external pick‐up points; 25% conventional‐not‐eligible; and 24% conventional‐not‐enrolled. Seventy‐four percent of all study participants expressed satisfaction with their HIV care. Those enrolled in DSD models were more likely to be satisfied, with an aOR of 6.24 (95% CI [3.18–12.24]) for external pick‐up point versus conventional‐not‐eligible and an aOR of 3.30 (1.95–5.58) for facility pick‐up point versus conventional‐not‐eligible. Conventional‐not‐enrolled clients were slightly but not significantly more satisfied than conventional‐not‐eligible clients (1.29, 0.85–1.96). Those seeking outside healthcare (crude OR 0.57, 0.41–0.81) or reporting more annual clinic visits (0.52, 0.29–0.93) were less likely to be satisfied. Conventional care participants reporting satisfaction with their current model of care perceived providers as helpful, respectful, and friendly and were satisfied with care despite long queues. DSD model participants emphasized ease and convenience, particularly not having to queue.

**Conclusions:**

Most adult ART clients in South Africa were satisfied with their care, but those enrolled in DSD models expressed slightly greater satisfaction than those remaining in conventional care. Efforts should focus on enrolling more eligible patients into DSD models, expanding eligibility criteria to cover a wider client base, and further improving the models’ desirable characteristics.

## 1 INTRODUCTION

Like many countries in sub‐Saharan Africa and globally [1, 2, 3, 4], South Africa has scaled up differentiated service delivery models (DSD) to improve HIV treatment coverage and outcomes, make treatment programmes more responsive to the needs of recipients of care [5, 6, 7] and increase service delivery efficiency [8, 9]. DSD models for antiretroviral therapy (ART) typically reduce the frequency of clinic visits, task shift to more suitable provider cadres and offer more convenient locations for service delivery [6]. Recent studies in South Africa and elsewhere have demonstrated equivalent or slightly better retention in care and viral suppression among eligible recipients of care enrolled in DSD, compared to those remaining in conventional care [10]. DSD models have also been shown to lower the time and financial costs of seeking ART for recipients of care [4, 11, 12] and to reduce the burden on service providers [13].

At the time of this study, South African HIV treatment adherence guidelines [9] specified up to three DSD models in which eligible recipients of ART could enrol. These were facility‐based medication pick‐up points, including lockers at the clinics that clients could access with individual codes; decentralized medicine delivery to contracted external pick‐up points, such as commercial pharmacies and community‐based lockers; and adherence clubs led by clinic staff, which could meet either at a healthcare facility or in the community. Some clinics also offered home‐based medication delivery. Clients not enrolled in a DSD model remained in conventional care. Not all facilities offered the option of all recommended models—some were limited to conventional care and only one or two other options. To be eligible for DSD model enrolment at the time of the study, ART clients had to be ≥18 years old, have been on ART for at least 12 months and have had two consecutive undetectable viral load test results [9].

A major goal of DSD models is to make ART delivery more “client‐centred,” improving recipients’ experiences with care and consequently increasing long‐term adherence and retention in care and potentially improving quality of life [5, 14, 15, 16]. Little is known, however, about how patient experiences in DSD models differ from those in conventional care. Recent studies showed high levels of self‐reported satisfaction ranging from 60% to 96% among ART clients in DSD models [17, 18], with higher satisfaction among those participating in out‐of‐facility models than those in facility‐based models [18]. These studies do not compare satisfaction among recipients of care in DSD models to that of recipients of care remaining in conventional care, however. Through the AMBIT project's SENTINEL survey [19], we made this comparison for a sample of facilities and recipients of care in South Africa.

## 2 METHODS

### 2.1 Overview and study sites

The first round of AMBIT's SENTINEL study was a cross‐sectional, mixed‐methods survey conducted among a convenience sample of adult (≥18 years old) ART clients in four provinces of South Africa from May to November 2021. The SENTINEL survey instrument included questions pertaining to participants’ satisfaction with HIV care, which are the results we report here. Other results pertaining to the costs of seeking care, time required for seeking care, clients’ preferences as to best and worst aspects of seeking care, providers’ views on DSD models, time‐and‐motion observations and resource utilization will be reported elsewhere.

Study sites for SENTINEL included 21 selected primary healthcare clinics in four districts of South Africa (Ehlanzeni, Ekurhuleni, King Cetshwayo and West Rand districts). The facilities were purposively selected because they had large ART client volumes based on the reported total remaining on ART (TROA), reflected a variation in settings (rural or urban) and demonstrated experience with DSD models for HIV treatment. Details about the study sites are provided in Table S1 and published elsewhere [19].

Conventional care at the time of the study entailed monthly clinic visits for individuals ineligible for DSD models or bimonthly clinic visits for those eligible for DSD models. At these visits, clients consulted with clinical providers, primarily nurses, and received 1‐ or 2‐month supplies of antiretroviral medications. Samples for viral load tests were drawn 6 months after treatment initiation, again at 12 months, and then every 12 months thereafter for those with suppressed viral loads. Clients with unsuppressed viral loads were counselled on medication adherence and asked to return within 3 months for a follow‐up assessment.

For this analysis, we classified the models of care that we encountered at the study sites into four categories: (1) internal pick‐up points, which included facility pickup points, facility‐based adherence clubs and facility‐based medication lockers; (2) external pick‐up points, which included off‐site (community) pick‐up points, home ART delivery and community‐based medication lockers; (3) conventional care for clients eligible for but not enrolled in a differentiated model; and (4) conventional care for patients not eligible for DSD models. At the time of study enrolment, both facility and community‐based adherence clubs had been scaled back significantly because of COVID‐19 response measures, and we did not encounter these models at most of the study sites.

### 2.2 Recruitment

At each study site, participants were approached consecutively as they arrived for routine clinic visits. ART clients were eligible to be enrolled in the study if they were eligible for DSD model enrolment (though not necessarily enrolled in a DSD model), had received at least one medication refill in their current model of care and were visiting the clinic for routine HIV‐related care rather than for some other purpose. At each study site, we enrolled up to 10 clients per model offered at that site, including DSD‐eligible and DSD‐ineligible clients receiving conventional care. As this was a descriptive study only, we did not calculate a target sample size but rather enrolled enough participants to provide sufficient information about each model of care, within the constraints of study time and resources.

Trained study interviewers consented and enrolled participants in the study following a referral from clinic staff. Eligible clients who provided written informed consent completed an interviewer‐administered, structured study questionnaire on the day of study enrolment. The study information sheet and questionnaire were available in English, Sesotho and isiZulu, which are most commonly spoken by recipients of care at the study sites. Data were collected on tablets using SurveyCTO v2.72.2 (https://www.surveycto.com/).

### 2.3 Survey content and outcomes

The survey was informed by a literature review and consultations with the research team. Overall satisfaction was assessed using a 5‐point Likert scale ranging from 1 (extremely dissatisfied) to 5 (very satisfied) for the question “How would you rate your overall satisfaction with the care that you receive at this facility?” for conventional care model patients and “How would you rate your overall satisfaction with your ART care now that you are in this model?” for those enrolled in DSD model care. Participants were also given an opportunity to elaborate on their responses regarding satisfaction with care received through open‐ended qualitative response options.

The primary outcome we report here is overall, self‐reported client satisfaction with care, stratified by model category. To assess overall satisfaction with HIV care received, we computed mean scores for overall satisfaction, then categorized the mean score as either “not satisfied” (score ≤3) or “satisfied” (score >3).

The survey also assessed self‐reported quality of care (QoC) using a 5‐point Likert scale. Components of QoC included questions related to provider attitudes, trust in providers, time spent with the provider, clinic administrative processes, healthcare access preferences and information received regarding HIV/ART. We conducted a factor analysis to identify reliable and interpretable scale items (Cronbach's alpha 0.70) [20]. Average scores were then calculated for overall self‐reported QoC and categorized into “low” QoC (score ≤3) and “high” QoC (score >3).

The survey also captured healthcare‐seeking factors that could affect client experiences. These included self‐reported time on ART, medication dispensing durations, whether the client had sought outside healthcare, additional diseases treated at the facility, the annual number of visit interactions and the number of missed visits annually. Facility characteristics collected included the setting of the facility (rural or urban) and facility size, which was based on TROA and was categorized into <2000 clients remaining on ART, 2000–4000 and >4000 [19]. The survey instrument is included as File S1.

### 2.4 Quantitative analysis

In this report, we first describe participant characteristics and healthcare‐seeking behaviour using proportions, frequencies, means with standard deviations and medians with interquartile ranges, as appropriate. We then use logistic regression to assess differences in overall satisfaction by DSD model participation, adjusting for self‐reported QoC, time on ART, seeking outside healthcare, additional diseases treated at the facility, dispensing duration, the annual number of clinic visits, missed visits, facility setting and facility size. We report adjusted odds ratios (aORs) and 95% confidence intervals (CI). We also conducted an age and gender‐stratified analysis, with results shown in the Supplementary Materials.

### 2.5 Qualitative analysis

For open‐ended questions, a codebook was developed in Microsoft® Excel using a combination inductive‐deductive approach. The coders first familiarized themselves with responses by reading the responses to each of the questions. Codes were refined and concepts were grouped or separated as needed based on the content of the responses. After the codebook was finalized, each question was reread and assigned all relevant codes. Additional codes were added to the codebook as they arose from the data. At the end of coding for each question, all responses coded as “other” were reviewed to determine if any were mentioned frequently enough to warrant a new code. The frequency of each code was calculated for each question overall and by the model of care assignment (conventional care, internal pick‐up point, external pick‐up point) for clients. The frequency of codes was used to identify major themes arising from the data. During analysis, responses within each model of care were reread for major themes to understand nuances specific to those model strata. Notable divergent views were identified and reviewed. Summary results and illustrative quotes are presented. Some quotations were edited slightly for grammar and clarity.

### 2.6 Ethics

The study protocol was reviewed and approved by the Human Research Ethics Committee of the University of Witwatersrand (M210241) and the Boston University IRB, protocol number H‐41402. All participants provided written informed consent. SENTINEL is registered on clinicaltrials.gov (NCT05886530) and the South African National Clinical Trial Registry (DOH‐27‐052023‐4669). National Health Research Database approval for conducting the survey was provided for each district.

## 3 RESULTS

### 3.1 Enrolment

**Figure 1:**
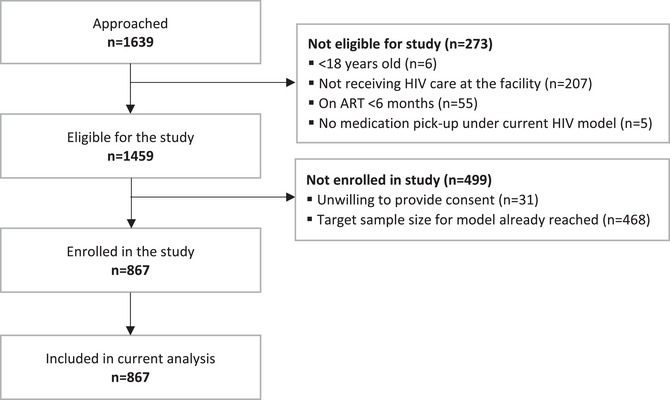
Recruitment, study eligibility and study enrolment among adults (≥18 years) at 21 primary healthcare clinics in four districts of South Africa.

We screened 1639 clients who were receiving HIV care at the study sites (Figure 1). Of these, 273 were not eligible to participate, 31 declined to participate and 468 were excluded because we had reached the enrolment target for the model of care in which they were enrolled (maximum of 10 per model per site). Screened clients excluded from the study were similar to those enrolled in age and gender. A total of 867 recipients of care were enrolled in the study.

### 3.2 Cohort characteristics

As shown in Table 1, 70% of the study cohort were female, with a median age of 39 years. More than three‐quarters (77%) were married or in a long‐term relationship, most (73%) had completed high school and over 50% were unemployed.

**Table 1:** Participant baseline socio‐demographic and HIV treatment characteristics (*n* = 867), by model category

Characteristic (*n*, %)	Conventional care—not eligible for differentiated service delivery models	Conventional care—eligible for differentiated service delivery models but not enrolled	Differentiated service delivery—facility pick‐up point	Differentiated service delivery—external pick‐up point	Total
*N* (row percentage)	216 (24.9)	209 (24.1)	206 (23.8)	236 (27.2)	867 (100)
***Socio‐demographic characteristics***					
Sex (female) (row percentage)	138 (63.9)	156 (74.6)	138 (67.0)	177 (75.0)	609 (70.2)
Age (years)					
Median (IQR)	39 (31–48)	37 (30–44)	40 (33–48)	40 (33.0–48.0)	39 (33–47)
16–24	21 (9.7)	16 (7.7)	20 (9.7)	7 (3.0)	64 (7.4)
25–35	58 (26.9)	72 (34.5)	42 (20.4)	52 (22.0)	224 (25.8)
35–49	86 (39.8)	92 (44.0)	100 (48.5)	127 (53.8)	405 (46.7)
50+	51 (23.6)	29 (13.9)	44 (21.4)	50 (21.2)	174 (20.1)
Marital status					
Married	38 (18.6)	45 (21.5)	49 (23.8)	54 (22.9)	186 (21.5)
In a relationship	116 (53.7)	118 (56.5)	112 (54.4)	134 (56.8)	480 (55.4)
Not in a relationship	62 (28.7)	46 (22.0)	45 (21.8)	48 (20.3)	201 (23.2)
Nationality (South African)	194 (89.8)	191 (91.4)	174 (84.5)	221 (93.6)	780 (90.0)
Highest level of education completed					
Primary school or less	39 (10.1)	35 (16.8)	30 (14.6)	40 (16.9)	144 (16.6)
High school	157 (72.7)	150 (71.8)	158 (76.7)	168 (71.2)	633 (73.0)
Post‐high school qualification	20 (9.3)	24 (11.5)	18 (8.7)	28 (11.9)	90 (10.4)
Employment status (formally, informally or self‐employed)	91 (42.1)	101 (48.3)	91 (44.2)	124 (52.5)	407 (46.9)
***HIV treatment characteristics (self‐reported)***					
Number of years on ART					
Median (IQR)	5.0 (2.3–8.0)	4.0 (1.8–7.0)	5.0 (3.4–8.5)	6.0 (3.0–10.0)	5.0 (3.0–8.5)
1–5 years	99 (45.8)	123 (58.9)	85 (41.3)	89 (37.7)	396 (45.7)
5–10 years	77 (35.7)	58 (27.8)	83 (40.3)	87 (36.9)	305 (35.2)
≥10 years	40 (18.5)	28 (13.4)	38 (18.4)	60 (25.4)	166 (19.1)
Any additional diseases treated at current facility (yes)	75 (34.7)	33 (15.8)	33 (16.0)	28 (11.9)	169 (19.5)
Seek healthcare from other providers outside of this facility (yes)	54 (25.0)	50 (23.9)	43 (20.9)	48 (20.3)	195 (22.5)
Number of combined nurse and medication collection visits per year					
Mean number of visits (SD)	7.3 (3.3)	6.3 (3.0)	3.7 (2.4)	2.3 (1.2)	4.8 (3.3)
1–2 visits	8 (3.7)	16 (7.7)	80 (38.8)	166 (70.3)	270 (31.1)
3–4 visits	35 (16.2)	41 (19.6)	58 (28.2)	55 (23.3)	189 (21.8)
5–12 visits	173 (80.1)	152 (72.7)	68 (33.0)	15 (6.4)	408 (47.1)
Number of medication collection‐only visits per year					
Mean number of visits (SD)	0.8 (1.8)	1.2 (2.1)	3.0 (2.3)	3.2 (2.5)	2.1 (2.4)
1–2 visits	189 (87.5)	164 (78.5)	79 (38.4)	83 (35.2)	515 (59.4)
3–4 visits	12 (5.6)	30 (14.4)	66 (32.0)	92 (39.0)	200 (23.1)
5–12 visits	15 (6.9)	15 (7.2)	61 (29.6)	61 (25.9)	152 (17.5)
Number of months of medication received at most recent visit					
Mean number of months (SD)	1.9 (1.0)	2.1 (0.7)	2.4 (0.7)	2.4 (0.5)	2.2 (0.8)
1 month	78 (36.1)	33 (15.9)	4 (2.0)	2 (0.8)	117 (13.6)
2 months	99 (45.8)	122 (58.7)	133 (65.8)	146 (61.9)	500 (58.0)
≥3 months	39 (18.1)	53 (25.5)	65 (32.2)	88 (37.3)	245 (28.4)
Number of self‐reported missed visits in the past year					
Mean number of missed visits, SD	2.4 (1.7)	2.1 (1.5)	1.9 (0.9)	1.7 (1.7)	2.1 (1.5)
None	114 (52.8)	136 (65.1)	142 (68.9)	180 (76.3)	572 (66.0)
1 visit	32 (14.8)	33 (15.8)	26 (12.6)	33 (14.0)	124 (14.3)
≥2 visits	70 (32.4)	40 (19.1)	38 (18.4)	23 (9.7)	171 (19.7)
Self‐reported quality of care					
High	198 (91.7)	178 (85.2)	184 (89.8)	216 (91.5)	776 (89.6)
Low	18 (8.3)	31 (14.8)	21 (10.2)	20 (8.5)	90 (10.4)
***Facility characteristics***					
Facility location					
Rural	103 (47.7)	98 (46.9)	102 (49.5)	80 (33.9)	383 (44.2)
Urban	113 (52.3)	111 (53.1)	104 (50.5)	156 (66.1)	484 (55.8)
Facility size—TROA					
<2000 TROA	63 (29.2)	59 (28.2)	69 (33.5)	47 (19.9)	238 (27.5)
2000–4000 TROA	92 (42.6)	90 (43.1)	85 (41.3)	95 (40.3)	362 (41.8)
>4000 TROA	61 (28.2)	60 (28.7)	52 (25.2)	94 (39.8)	267 (30.8)

Abbreviations: ART, antiretroviral therapy; DSD, differentiated service delivery; IQR, interquartile range; SD, standard deviation; TROA, total remaining on ART.

The 867 participants enrolled in the study were evenly distributed among the four categories of models of care. There were no important differences between service delivery models in terms of socio‐demographic characteristics.

The median self‐reported time on ART was 5 or 6 years for all participants except those eligible for but not enrolled in DSD models, who had a median of 4 years on ART and a higher proportion who had been on treatment for <4 years than did the other models. A fifth of study participants reported being treated for additional diseases at the facility, including 35% of those not eligible for DSD enrolment. Just over one‐fifth (22%) reported seeking healthcare outside their current facility, with no differences among models.

The number of clinic visits and medication pick‐up visits reported by participants over the previous year varied by service delivery model. Most (70%) participants who were enrolled in external pick‐up points attended 1–2 nurse and medication collection visits annually, compared to only 38.8% of those enrolled in facility pick‐up points. A further 54% of clients who used facility pick‐up points attended 3–6 nurse and medication collection visits annually. In contrast, most (77%) of those enrolled in conventional care attended at least five and as many as 12 nurse and medication collection visits annually. Patients enrolled in facility or external pick‐up points were more likely to receive 2 or more months of medication per refill rather than just one. Self‐reported missed visits were higher among those enrolled in conventional care, with a quarter of the participants missing two or more visits annually compared to 18% and 10% among those enrolled in facility pick‐up points and external pick‐up points, respectively. A large majority of study participants (90%) reported that they perceived the QoC they received to be high, regardless of the model of care.

A higher proportion (56%) of study participants were enrolled from facilities in urban areas than in rural areas. Almost a third (31%) were enrolled from large facilities with a TROA exceeding 4000 clients on ART, while more than a quarter originated from relatively small facilities with fewer than 2000 clients on ART.

### 3.3 Quantitative results: client satisfaction with HIV care

**Figure 2:**
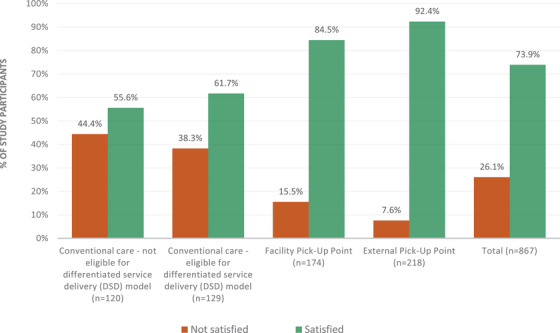
Client satisfaction with HIV care in differentiated service delivery models compared to conventional care (*n* = 867).

Overall, 74% of participants reported high satisfaction with HIV care (Figure 2 and Figure S1). A large majority enrolled in facility pick‐up points (85%) and external pick‐up points (92%) expressed satisfaction with their HIV care. A substantial minority (41%) of clients remaining in conventional care reported dissatisfaction with the care they received, including 38% among those eligible and 44% among those not eligible for DSD enrolment.

**Figure 3:**
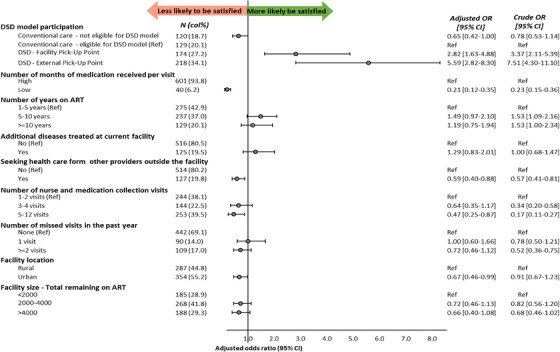
Crude and adjusted* odds ratios of client satisfaction among differentiated service delivery model and conventional care study participants (*n* = 861). *Adjusted for perceived quality of care, time on ART, seeking outside healthcare, additional diseases treated at the facility, dispensing duration, annual number of clinic visits, annual number of missed visits, facility location and facility size.

Figure 3 presents crude and aORs of participant baseline factors associated with self‐reported satisfaction with care. Consistent with the descriptive results in Figure 2, by far the largest predictor of greater satisfaction was enrolment in one of the DSD models, particularly the external pickup point model (aOR 5.59; 95% CI 2.82–11.10). Figure 3 indicates that those attending more clinic visits annually (aOR 0.47 for 5–12 visits vs. 1–2 visits; 95% CI: 0.25–0.87), those enrolled in facilities in urban areas (aOR 0.67; 95% CI 0.46–0.99) and those seeking outside healthcare (aOR 0.59; 95% CI 0.40–0.88) were somewhat less likely to report satisfaction with their current HIV care. As might be anticipated, low perceived QoC was associated with low satisfaction with HIV care (aOR 0.21; 95% CI 0.12–0.35). No other factors were significantly associated with client satisfaction.

When the analysis was restricted to include only participants eligible for DSD models (including those enrolled and those eligible but not enrolled), the results remained consistent (Figure S2).

Stratification by age and gender did not change our primary findings. Satisfaction with care was higher among more experienced male clients, however (aOR 2.99 for 5–10 years vs. 1–2 years; 95% CI: 1.33–6.70). Female participants from high‐volume sites were less likely to be satisfied (aOR 0.52 for 2000–4000 TROA vs. <2000 TROA; 95% CI: 0.30–0.90; aOR 0.50 for >4000 TROA vs. <2000 TROA; 95% CI: 0.27–0.93), as were older clients (aOR 0.60 for 2000–4000 TROA vs. <2000 TROA; 95% CI: 0.35‐1.00; aOR 0.50 for >4000 TROA vs. <2000 TROA; 95% CI: 0.28‐0.88) (Figures S3 and S4).

### 3.4 Qualitative responses

In open‐ended responses, most of the 41% of study participants enrolled in conventional care, whether eligible for DSD participation or not, who indicated dissatisfaction with care described frustration with long waiting times at the clinics and attributed this to overcrowding, staff shortages and/or slow staff (Table 2). A few reported previously being enrolled in a DSD model and rated the current services in conventional care as poor in comparison. Themes were not different between those in conventional care who were eligible for DSD and those who were not eligible.

**Table 2:** Participants’ comments regarding satisfaction

Model of care	Overall satisfaction	Client (sex, age in years)	Illustrative comments
Conventional care (eligible for differentiated service delivery or not)	Satisfied	Male, 61	“Although the waiting times are long, the nurses are friendly and they help us in managing our health.”
		Female, 58	“I'm happy with their service. Nurses treat me well and always encourage me to not miss appointments.”
		Male, 62	“The facility is closer to me and I always get help when I visit the clinic. The staff is very efficient.”
		Female, 54	“The staff is friendly. Even when you miss an appointment, they understand.”
	Not satisfied	Female, 48	“Long hours waiting to receive medications and coming every month for collections.”
		Male, 33	“Shortage of staff and long hours to be assisted.”
		Female, 41	“The waiting time is too long since I was taken out of facility pick‐up point, so I am not satisfied as I wasn't able to go to work due to long waiting hours.”
		Female, 36	“The clinic is small and has few staff which makes long waiting hours.”
External pick‐up point	Satisfied	Male, 38	“The flexibility in collecting the medication from the Pele Box (medication locker) allows me to collect after hours and there are no days missed at work.”
		Female, 34	“It is near me, and I don't come every month and I can send someone to collect only on my behalf.”
		Male, 36	“At the external pick‐up points the queues are always short it's very convenient as I can go at any time without worrying that I need to go early for collecting meds like at the clinic.”
		Female, 20	“I am a student so I am happy with the model when they deliver my medication at home as I get to study and do other things, unlike coming to the facility on a monthly basis.”
	Not satisfied	Female, 57	“I am not satisfied because I felt comfortable coming to the clinic.”
		Female, 46	“I don't get the pin (for the medication locker) to collect the medication and this is the second time that its happening and I won't be able to go to work.”
		Male, 43	“The external pick‐up point is busy.”
Facility pick‐up point	Satisfied	Female, 32	“When we come to the clinic we don't wait for too long for our treatment and we have the option to send someone to collect it for you if you don't have time to come to the clinic.”
		Female, 35	“Because I come less to the clinic to collect medication than coming every month.”
		Female, 50	“Because it makes my life easier I don't have to wait too long at the facility since I was upgraded to the facility pick‐up and I am happy in such a way that I would rate them 100%.”
		Female, 21	“Before being in the club I would have to be absent at school for clinic visit but now the club is flexible with dates and time.”
	Not satisfied	Female, 62	“The time I spend at the clinic is too long compared to the time I used to pick outside the clinic. I was brought back because of my BP and I now pick‐up at the clinic.”
		Male, 28	“I have to wait long hours in the queues before I get assistance. I miss some piece jobs because of waiting for a long time in the clinic.”
		Male, 19	It was nice when collecting outside now they brought me back here, always waiting in long queues.”
		Female, 53	“The facility is far from home. It takes long walking distance to get to the clinic on scheduled days.”

Among those who reported that they were satisfied, an emerging theme was that the providers treated them well and took care of them. Several also described a short wait or quick service at the clinic and being able to see a provider or pick up their medication without having to wait long. Others reported inconsistent wait times, some short and some long.

Study participants who obtained their medication from external pick‐up points and those who utilized facility pick‐up points cited short waiting times, lack of long queues, quick service and the option to have someone else collect their medication as factors contributing to their satisfaction. Those using external pick‐up points found the location convenient, allowing them to save time and avoid disruptions to their daily routines. Facility pick‐up point clients appreciated the convenience of receiving multiple months of medication at once and expressed satisfaction with the consistent availability of medications at the clinic. Some clients using lockers faced challenges with accessing PIN numbers. Dissatisfaction among clients utilizing facility or external pickup points, which was rare, primarily stemmed from long queues at pickup points.

## 4 DISCUSSION

In this survey of adult ART clients in South Africa, most (74%) participants across both conventional care and DSD reported being satisfied with their care and most (90%) said that they were receiving a high QoC. Participants in DSD models were more likely to report a high level of satisfaction with the care received, however, than were their counterparts remaining in conventional care. As might be anticipated, participants who perceived their QoC to be low were less likely to be satisfied with care. In view of the importance of perceived QoC and of satisfaction in sustaining engagement in care, confirming that clients enrolled in lower‐intensity models that invest fewer resources (e.g. clinical consultations) in each individual are as or more satisfied than they were in conventional care is an important finding.

Our study included a population of clients who were eligible for DSD models but not enrolled. There were likely a number of reasons for the non‐enrolment, including clients’ preference to remain in routine care, providers’ judgement as to patient readiness for a lower intensity model, the availability of a suitable model at the facility in question and/or South Africa's requirement that a client has a national identification number for DSD model enrolment [21, 22, 23]. Some clients in this group may also have been back‐referred to conventional care after DSD model enrolment due to missed visits or other concerns. Future research is needed to understand the reasons for non‐enrolment of eligible clients, particularly in light of the slightly lesser level of satisfaction among those not enrolled.

Our findings are consistent with those of previous studies assessing overall satisfaction with routine HIV care in high HIV burden settings [24, 25, 26]. A study in Eswatini found that 96% of study participants were satisfied with their current ART delivery model, and 90% recommended it to others [17]. Recipients of care from Khayelitsha in Cape Town, South Africa, also reported generally positive experiences with DSD model care [27], and 64.2% of those enrolled in DSD model care in Uganda reported high satisfaction with the treatment [18]. These prior studies did not compare satisfaction with DSD model care with conventional care [17, 18, 28]. This study contributes to the knowledge base by providing this comparison, finding that those in DSD models are more likely to express satisfaction with their care.

Qualitative results from the current study corroborate the quantitative findings and indicate that ART clients consider existing DSD models to be more convenient. They reported shorter waiting times and quicker service delivery, especially among those collecting their treatment at external pick‐up points. Convenient and accelerated service delivery are considered important drivers of service utilization [27]. DSD enrolees also lauded this aspect of health service delivery in DSD models in Khayelitsha, Cape Town and Eswatini [17, 28]. While the proportion of study participants reporting dissatisfaction was minimal, the issues reported by participants as causes of dissatisfaction in the qualitative responses align with what has previously been reported by public sector clients.

We note that previous studies in South Africa have revealed high satisfaction levels with conventional (undifferentiated) HIV care despite frequent complaints. Complaints about long waiting times and other characteristics associated with poor quality care are relatively common in the public health sector [26, 29]. To explain this apparent contradiction, we note that one of the key factors that determines clients’ satisfaction is their expectations regarding services [30, 31]. An entrenched perception of poor service delivery is associated with public healthcare provision in South African public healthcare [32]. Long waiting times, inadequate infrastructure for queuing, insufficient privacy during clinical consultations and poor attitudes from health providers are common complaints [32, 33]. As a result, we speculate that most clients expect poor service quality [30, 31, 34]. They may thus report feeling satisfied regardless of poor quality, as they anticipated nothing better. Exploring expectations, in addition to actual experiences, and interpreting self‐reported satisfaction with care when expectations are very low, may, therefore, be another valuable pathway for future research. In a recent study in Zambia, trained ART patients were more likely to report suboptimal care during exit interviews than untrained patients. The training may have increased their expectations of care and empowered them to be more critical in their assessment of satisfaction with the quality of services received [35].

Our study had several limitations. We conducted a cross‐sectional study that provided a snapshot of self‐reported client satisfaction at a single point in time. Clients’ experiences may change over time. Our sample was limited to a small number of ART clinics per model and a small number of facilities and districts; geographic generalizability is unclear. Our results reflect the same survival bias as any cross‐sectional survey; only those currently in care were enrolled. We thus have no response from those no longer in care who may have a different perspective, especially if negative experiences hindered retention in care.

We also know that enrolment in DSD models is unlikely to be random. If there is an underlying association between DSD model enrolment and satisfaction with care, such that clients who enrol in DSD models would have expressed greater satisfaction with their care even in the absence of DSD, our analysis may overstate the contribution of DSD models to client satisfaction. The opposite may also be true, however: clients least satisfied in conventional care may already have self‐selected into DSD models and express greater satisfaction as a result of their opportunity to make a change. It is also possible that at the time of the survey, some study participants had already switched models of care due to dissatisfaction with a previous model, independently increasing their satisfaction with their current model simply because it is different from one they previously disliked. Similarly, as DSD models are commonly perceived as having more benefits, individuals not enrolled in these models may experience additional dissatisfaction that stems from the perception of missing out on these benefits, rather than from their QoC per se. The inability to know whether participants had intentionally switched models prior to the time of the survey could have led to misclassification bias, and future studies that follow patients over longer time horizons will be helpful to better understand their patterns of healthcare utilization and the trajectories of their engagement in care.

Finally, data collection for the study was carried out soon after COVID‐19 restrictions were eased after South Africa's National State of Disaster was lifted. The COVID‐19 response included restrictions on physical movement and changes to clinic operating hours. Healthcare access was severely affected by these restrictions and by the general fear of visiting health facilities at the time, and client experience may reflect these conditions [36]. Alternatively, contingency measures to ease access, such as clinics extending ART prescriptions and dispensing duration [37], may have improved the client experience. Future rounds of SENTINEL survey data collection will potentially mitigate this effect and facilitate the comparison of data over time.

## 5 CONCLUSIONS

Most adult ART clients in South Africa were satisfied with the service delivery model in which they were enrolled, whether conventional or differentiated, but those participating in DSD models expressed greater satisfaction than did those receiving conventional care. For policymakers, our survey provides reassurance that offering services in new ways, such as those exemplified by internal and external dispensing points, are considered by users to be as good as, or better than, conventional care. These results, when considered alongside publications that report equivalent or better clinical outcomes [10] and equivalent or lower costs [38], suggest that policymakers and programme managers should prioritize enrolling more eligible patients in existing DSD models, expanding eligibility criteria to cover more of the client population and further improving the valued characteristics of the existing models.

## COMPETING INTERESTS

The authors declare that they have no competing interests.

## AUTHORS’ CONTRIBUTIONS

IM, SR, AH and SP conceptualized the study. IM, SR, AH, SP, NL, VN and CH contributed to instrument design. IM, VN, JLK and NS analysed the data. IM, NS and SR drafted the manuscript. All authors reviewed and approved the final manuscript.

## FUNDING

Funding for the study was provided by the Bill & Melinda Gates Foundation through OPP1192640 to Boston University.

## DISCLAIMER

The funders had no role in study design, data collection, analysis, or interpretation of data or in the writing of this manuscript.

## Supporting information


**File S1**. SENTINEL survey instrument

**Figure S1**. Responses to client satisfaction questions

**Figure S2**. Adjusted analysis for stable participants

**Figure S3**. Age stratified analysis

**Figure S4**. Gender stratified analysis

**Table S1**. Characteristics of the SENTINEL study sites

## Data Availability

Disidentified data will be posted in a public repository following closure of the study protocol with the supervising ethics committees; the corresponding author can be contacted for details.
