# Supplementary material for: Satisfaction with service delivery among HIV treatment clients enrolled in differentiated and conventional models of care in South Africa: a baseline survey
Source: J Int AIDS Soc. 2024 Mar 25;27(3):e26233. doi: 10.1002/jia2.26233 (PMC10963588; doi:10.1002/jia2.26233)
Supplement: Supplementary file 1 — File S1. SENTINEL survey instrument [file JIA2-27-e26233-s004.pdf]

Survey ID

Sentinel-South Africa Patients' Survey

Surveyor ID ____________________________________ Facility name ______________________________

Date (DD/MM/YYYY) ______________________________ Location within facility ______________________

Surveyor notes:

Introduction

Surveyor: Read the following statement. Please repeat the statement translated into the local language based on primary languages.

"Thank you for agreeing to participate in this survey. My name is ______. I will be asking you the questions. Most of the questions require that you select one of the options as your answer, although some questions you can select all the answers that apply. I will specify the options and instructions for you as I ask each question. If your answer is not one of the specified options please tell me and I will write your answer down. Please feel free to tell me whatever you are comfortable sharing. You should also remember that you do not have to share anything that you are not comfortable sharing and that you can stop this interview at any time without any risk to your rights or treatment and care. There are no right or wrong answers, so please be honest and help us to understand what is true for you and your community. Are you ready to begin?

Part 1.

Surveyor: "I'm going to start by asking you some basic questions about who you are, where you live, and your education and employment."

Q#	QUESTION	RESPONSES
**Respondent demographics and socio-economic status**		
1.	Patient study ID	
2.	Patient file number	
3.	What is your gender?	0= Male<br>1= Female<br>2= Other
4.	What is your nationality/country of origin?	1= South Africa<br>2= Botswana<br>3= Lesotho<br>4= Mozambique<br>5= Malawi<br>6= Namibia<br>7= eSwatini<br>8= Zambia<br>9= Zimbabwe<br>10=Tanzania<br>11=Burundi<br>12= Other African country (specify)<br>13= Other (specify)
5.	If you are not South African, how long have you lived in South Africa?	1= < 1 yr<br>2= 1-2 years<br>3= 2-5 years<br>4= >5 years<br>5= Seasonal work

Q#	QUESTION	RESPONSES
6.	How old were you at your last birthday?	Age (years)<br>Don't know
7.	What is your marital status?	1= Never married<br>2= Married (customary/traditional or legal/civil)<br>3= Divorced<br>4= Separated<br>5= Widowed
8.	Is there someone who you have a relationship with and who you call your partner?	1= No<br>2= Yes
9.	Do you currently live with your husband/wife or your partner?	1= No<br>2= Yes, married or living together
10.	Do you think of the house you currently live in as your main house?	1= Yes<br>2= No, my main house is somewhere else in South Africa<br>3= No, my main house is in another country
11.	Do you know how to read and write?	1= No<br>2= Yes – read and write<br>3= Yes – read only
12.	What was the highest level of school that you completed?	1 = No schooling<br>2= Primary<br>3= Secondary<br>4= Certificate/Diploma/ Post-secondary<br>5= Graduate degree
13.	What is your occupation?	1= Farming (my own or my family's farm)<br>2= Farm worker (someone else's farm)<br>3= Domestic worker or carer (paid)<br>4= Informal sector job (not farming or domestic) (e.g. trader, day service provider)<br>5= Formal sector job (salaried)<br>6= Household work and/or childcare (my own house, not paid)<br>7= Unemployed but looking for work<br>8= Student or trainee<br>9= Retired<br>10= Other (specify)
14.	Do you have any living children?	0= No<br>1= Yes
15.	How many living children do you have?	Specify number
16.	How many adults age 15 or older are living in your household, including yourself? By "living in," I mean they spend most nights of the week sleeping in your household.	Specify number
17.	How many children age 14 or younger are living in your household, including yourself? By "living in," I mean they spend most nights of the week sleeping in your household.	Specify number
18.	Do you have electricity in your house?	0= No<br>1= Yes
19.	Do you have access to piped water?	0= No<br>1= Yes – to house<br>2= Yes – community tap/pipe
20.	Do you or the people in your household go without food often, sometimes, seldom, never?	1= Never<br>2= Seldom

Q#	QUESTION	RESPONSES
		3= Sometimes<br>4= Often
21.	Do you or does anybody in your household, currently receive any support or grant from the government? Tick all that apply)	0= No<br>1= Child grant<br>2= Partial disability / illness grant / temporary grant<br>3= Pension grant<br>4= Disability grant<br>5= Unemployment grant<br>6= Other (specify)
22.	If a person in your household became ill and 100 Rands was needed for treatment or medicines, would you say it would be very easy, easy, difficult, or very difficult to find the money?	1= Very difficult<br>2= Difficult<br>3= Easy<br>4= Very easy
**Healthcare access and cost**		
23.	How long have you been coming to this facility for any kind of HIV treatment?	Months/years
24.	How long have been taking ART?	Months/years
25.	Which diseases are you currently being treated for at this facility in addition to HIV? (Tick all that apply)	0= No other diseases<br>1= TB<br>2= Diabetes<br>3= Hypertension<br>4= Asthma<br>5= Mental health<br>6= Malaria<br>7= Other (specify)
26.	What is the main reason for you coming to this facility today?	1= Scheduled HIV treatment visit<br>2= Unscheduled HIV treatment visit<br>3= HIV medication refill only<br>4= Other (specify)
27.	In the past 12 months have you sought health care from any other health care provider outside this facility? (Tick all that apply)	0= No<br>1= Hospital<br>2= Private doctor<br>3= Traditional healer<br>4= Community health worker<br>5= Local NGO/FBO<br>6= Other (specify)
28.	How do you usually get to the clinic? (Tick all that apply)	1= Walk<br>2= Mini-bus/common taxi<br>3= Own car<br>4= Meter taxi/Uber/Taxify<br>5= Brought by family/friends in their vehicles<br>6= Other (specify)
29.	How long does it take you to get to the clinic? (One way – from home to the clinic)	Hours/minutes one way
30.	What expenses/costs do you incur for each clinic visit? (Tick all that apply)	0= No costs<br>1= Transport<br>2= Loss of income due to missing work<br>3= Child care<br>4= Food/drinks<br>5= Other (specify)
31.	If you pay for transport, please estimate how much does public transport cost you in Rands each time you visit the clinic (Return trip – to the clinic and back home)	Amount in Rands

Q#	QUESTION	RESPONSES
32.	Please estimate how much does unpaid time off work cost you in Rands each time you visit the clinic	Amount in Rands
33.	Have there been occasions where you missed your facility visits in the past year by more than 2-3 days?	0= No<br>1= Yes
34.	If so, why did you miss the visit?	1= Forgot pick up date<br>2= Ill health<br>3= Nobody else to go for me<br>4= Buddy forgot<br>5= Buddy unwell<br>6= No money for transport<br>7= Could not leave work<br>8= Afraid HIV status will get known<br>9= Other (specify)
**Questions for patients in standard care**		
35.	For your HIV treatment, how many visits to this clinic where you both see a nurse and collect your medication do you attend per year?	Number
36.	For your HIV treatment, how many visits to this clinic where you do <u>not</u> see a nurse but do collect your medication do you attend per year?	Number
37.	How long does it take total, on average for each HIV clinic visit where you see a nurse and pick up medications (counting from when you arrive at the clinic to when you leave)?	Hours and minutes
38.	How long does it take total, on average for each ART medication pick-up (visits where you only pick up medications, do not see a nurse)?	Hours and minutes
	Please explain your answer (satisfaction rating)	Open ended
39.	How would you rate your overall satisfaction with the care that you receive at this facility?	1= Extremely dissatisfied<br>2= A little dissatisfied<br>3= Neither satisfied nor dissatisfied<br>4= Satisfied<br>5= Very satisfied
40.	Please explain your satisfaction rating above	Open ended
41.	Please state how much you agree or disagree with the following statements	These are all Likert scales 1-5 (strongly disagree, mildly disagree, neither agree or nor disagree, mildly agree, strongly agree, no response/don't know)
a.	I receive enough information about HIV and ART	
b.	Nurses and other clinicians at this facility spend enough time with me	
c.	When I arrive, the clinic can find my file promptly	
d.	My laboratory test results are available when I come back for them	
e.	People at this clinic are always nice and friendly	
f.	I trust the healthcare providers I see at this clinic	
g.	I would like to come to this clinic more often than my current appointments	
42.	How could HIV services in this facility be improved? (select all that apply)	☐ More staff<br>☐ More information provided by staff<br>☐ Better, more polite, or friendlier staff attitude/manner<br>☐ Better location<br>☐ Open different days<br>☐ Open different times of day

Q#	QUESTION	RESPONSES
		☐ Open outside of work hours<br>☐ Shorter waiting time<br>☐ More counselling when there are problems<br>☐ More counselling overall<br>☐ Less counselling<br>☐ Being able to pick up ARVs at different and more convenient sites<br>☐ Being able to have someone else pick up your ARVs<br>☐ Having somebody to support you take your ARVs<br>☐ Tracing when missed an appointment<br>☐ Reminders via phone<br>☐ More months of ARVS given at each visit<br>☐ Fewer months of ARVS given at each visit<br>☐ Better access to a nurse or clinic staff<br>☐ Treatment/support for other illnesses(specify)<br>☐ Other (specify and elaborate)
**Questions for patients in DSD models**		
43.	When were you first enrolled in Model X?	Month/year
44.	Did you ask to be enrolled in Model X?	Yes/no
45.	Did you have to provide consent to be enrolled in Model X?	0= No<br>1= Yes – written consent<br>2= Yes – verbal consent<br>99= Not sure/don't know/can't remember
46.	Were you given a choice about joining Model X?	0= No<br>1= Yes<br>99= Not sure/don't know/can't remember
47.	Were you happy to be enrolled in a DSD model?	0= No<br>1= Somewhat<br>2= Yes<br>3 = Neither happy nor unhappy (did not care)
48.	How would you rate your overall satisfaction with your ART care <u>before</u> you started Model X?	1= Extremely dissatisfied<br>2= Not satisfied<br>3= Neither satisfied nor dissatisfied<br>4= Satisfied<br>5= Very satisfied
49.	How would you rate your overall satisfaction with your ART care now that you are in Model X?	1= Extremely dissatisfied<br>2= Not satisfied<br>3= Neither satisfied nor dissatisfied<br>4= Satisfied<br>5= Very satisfied
50.	Please explain your satisfaction rating for Model X	Open ended
51.	Please state how much you agree or disagree with the following statements pertaining to Model X	These are all Likert scales 1-5 (strongly disagree, mildly disagree, neither agree or nor disagree, mildly agree, strongly agree, no response/don't know)
a.	I receive enough information about HIV and ART	
b.	Nurses and other clinicians at this facility spend enough time with me even though I'm in another model	

Q#		QUESTION	RESPONSES
	c.	When I arrive, the clinic can find my file promptly	
	d.	My laboratory test results are available when I come back for them	
	e.	People at this clinic are always nice and friendly	
	f.	I trust the healthcare providers I see at this clinic	
	g.	People who manage my model of care are always nice and friendly	
	h.	I trust the healthcare providers who manage my model of care	
	i.	I would like to come to this clinic more often than my model of care allows	
52.		For your HIV treatment, how many visits to this clinic where you both see a nurse and collect your medication do you attend per year?	Number/year
53.		For your HIV treatment, how many visits to this clinic where you do <u>not</u> see a nurse but do collect your medication do you attend per year?	Number/year
54.		How many out-of-facility HIV events do you attend per year? By "event" we mean anything involving your treatment that is not at this facility, including club meetings, picking up medications outside the clinic, etc.	Number/year
55.		If you have a question about your HIV treatment, what would you most likely do?	1= Make a special visit to this clinic<br>2= Wait and ask during a regular visit to this clinic<br>3= Wait and ask during a regular out-of-facility HIV event<br>4= Other (specify)
56.		How do you usually get to the out-of-facility Model X events (Tick all that apply)	1= Walk<br>2= Mini-bus/common taxi<br>3= Motor bike taxi<br>4= Hired taxi<br>5= Brought by family/friends in their vehicles<br>6= Other (specify)
57.		How long does it take you to travel to the out-of-facility Model X events? (One way – from home to the clinic)	Hours and minutes (one way)
58.		What expenses/costs do you incur for each out-of-facility Model X event? (Tick all that apply)	0= No costs<br>1= Transport<br>2= Lost income due to missed work<br>3= Child care<br>4=Food/drinks<br>5= Other (specify)
59.		If you pay for transport, please estimate how much does transport cost you in Rands each time you make an out-of-facility Model X visit.<br>(Return trip – to the clinic and back home)	Amount in Rands
60.		Please estimate how much income (wages or salary) you lose in Rands each time you attend a Model X event.	Amount in Rands
61.		How long does it take total, on average for each out-of-facility Model X event	Hours and minutes, including travel time and time at event
62.		How would you rate your overall satisfaction with your out-of-facility Model X events?	1= Extremely dissatisfied<br>2= Not satisfied<br>3= Neither satisfied nor dissatisfied

Q#	QUESTION	RESPONSES
		4= Satisfied<br>5= Very satisfied
63.	Have you been referred back to regular (standard of care) HIV services by the Model X staff for any reason? (Tick all that apply)	0= No<br>1= Ill health<br>2= Missed a visit<br>3= Missed ARV doses<br>4= Blood draw<br>5= Screened positive for TB<br>6= Diabetes complication<br>7= Hypertension complication<br>8= Don’t know why
64.	Have there been occasions in the past year where you missed your out-of-facility Model X event by more than 2-3 days?	0= No<br>1= Yes
65.	If so, why did you miss the visit?	1= Forgot pick up date<br>2= Ill health<br>3= Nobody else to go for me<br>4= Buddy forgot<br>5= Buddy unwell<br>6= No money for transport<br>7= Could not leave work<br>8= Afraid HIV status will get known<br>9= Other (specify)
66.	Please state how much you agree or disagree with the following statements	These are all Likert scales 1-5 (strongly disagree, mildly disagree, neither agree or nor disagree, mildly agree, strongly agree, no response/don’t know)
a.	I am concerned about transport cost to get to the clinic	
b.	I am concerned about transport cost to get to my model events (e.g. club meetings, medication pickups)	
c.	I cannot take time off work to get to the appointments	
d.	I am unsure about how the DSD model works	
e.	I don’t receive enough information about HIV and ART	
f.	I have to wait for a long time to receive care	
g.	I am concerned about safely carrying and storing ARVs at home	
h.	I am concerned about other people finding out that I am HIV positive	
i.	I am concerned about having to interact with other patients in my treatment model	
j.	I am concerned about the quality of care in my treatment model	
k.	I have other concerns about my HIV treatment or model of treatment delivery (specify)	
l.	I have no concerns about my HIV treatment or model of service delivery	
m.	I have trouble taking time off work to get to clinic visits	
n.	I have trouble taking time off work to get to model events	

Q#		QUESTION	RESPONSES
	o.	I prefer to come to the clinic as few times per year as possible	
	p.	I'd like to come to the clinic regularly, at least once every 3 months	
	q.	I like being able to connect with other HIV-positive patients at the clinic	
	r.	I like being able to connect with other HIV-positive patients in my model of care	
67.		Would you recommend Model X to another patient who is on ART treatment?	0=No<br>1=Yes
68.		Why or why not?	Open ended
69.		How could services on this Model X model be improved? (select all that apply)	☐ More staff<br>☐ More information provided by staff<br>☐ Better, more polite friendlier, staff attitude<br>☐ Better location for model events<br>☐ Events on different days<br>☐ Events at different times of day<br>☐ Events outside of work hours<br>☐ Shorter waiting time<br>☐ More counselling when there are problems<br>☐ More counselling overall<br>☐ Less counselling<br>☐ Being able to pick up ARVs at different and more convenient sites<br>☐ Being able to have someone else pick up your ARVs<br>☐ Having somebody to support you take your ARVs<br>☐ Contacting you when you miss an appointment<br>☐ Reminders via phone or SMS<br>☐ More months of ARVs given at each visit<br>☐ Fewer months of ARVs given at each visit<br>☐ Better access to a nurse or clinic staff<br>☐ Treatment/support for other illnesses (specify)<br>☐ Other (specify and elaborate)
70.		In conclusion, what is the best thing for you about Model X?	Open ended
71.		And what is the worst thing for you about Model X?	Open ended

Surveyor: Please thank the participant for their time and ask if they have any additional questions about the study.

Surveyor initials ___________

Supervisor initials ___________

Date reviewed by supervisor ___________
