# Supplementary material for: Satisfaction with service delivery among HIV treatment clients enrolled in differentiated and conventional models of care in South Africa: a baseline survey
Source: J Int AIDS Soc. 2024 Mar 25;27(3):e26233. doi: 10.1002/jia2.26233 (PMC10963588; doi:10.1002/jia2.26233)

Figure S1. Responses to client satisfaction questions among participants in HIV care in DSD models compared to conventional care (n=867)


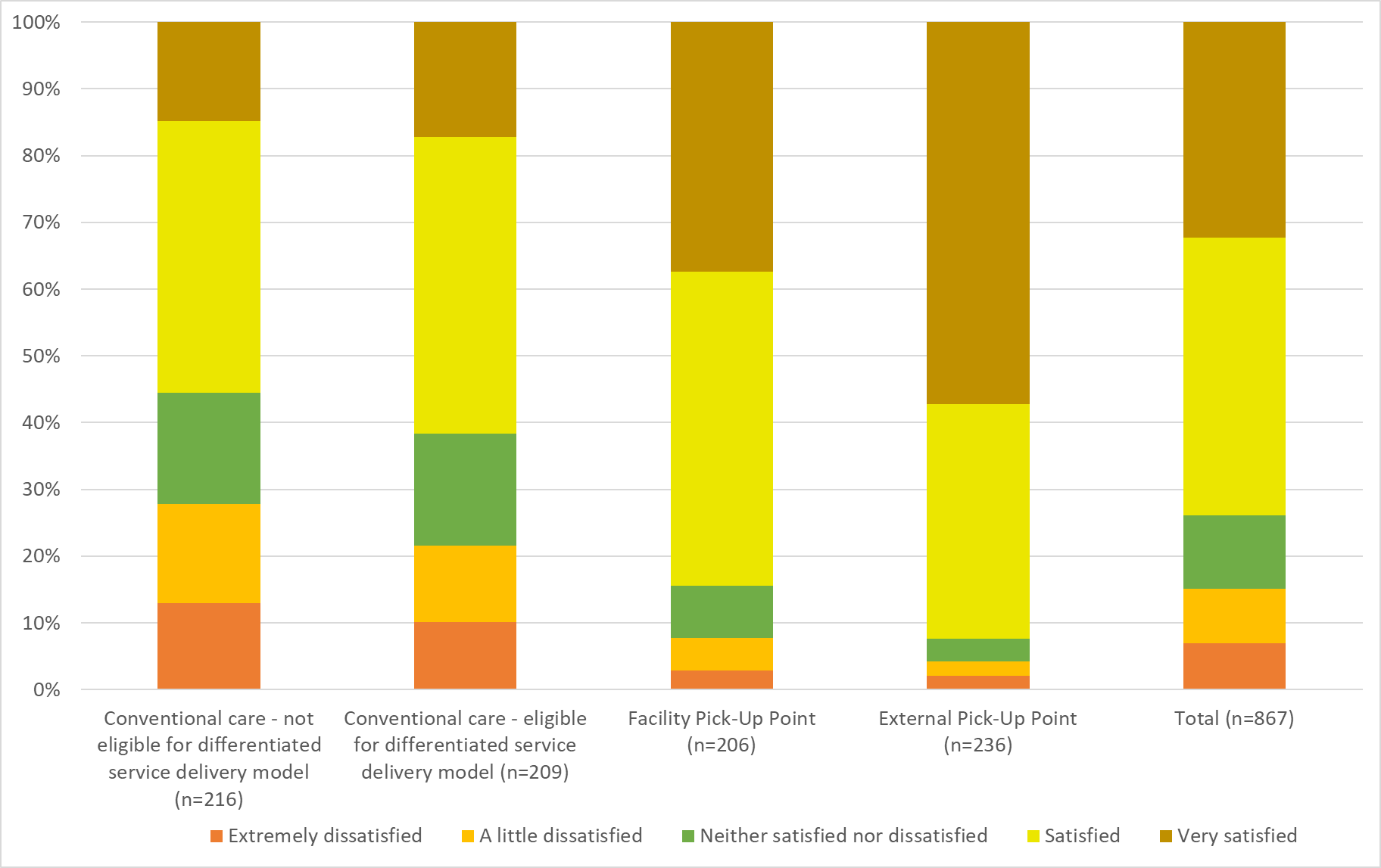

Supplement: Supplementary file 2 — Figure S1. Responses to client satisfaction questions [file JIA2-27-e26233-s006.docx]
