# Supplementary material for: Satisfaction with service delivery among HIV treatment clients enrolled in differentiated and conventional models of care in South Africa: a baseline survey
Source: J Int AIDS Soc. 2024 Mar 25;27(3):e26233. doi: 10.1002/jia2.26233 (PMC10963588; doi:10.1002/jia2.26233)
Supplement: Supplementary file 3 — Figure S2. Adjusted analysis for stable participants [file JIA2-27-e26233-s002.docx]

Figure S2. Crude and adjusted* odds ratios of client satisfaction for DSD model and conventional care eligible study participants (n=651)


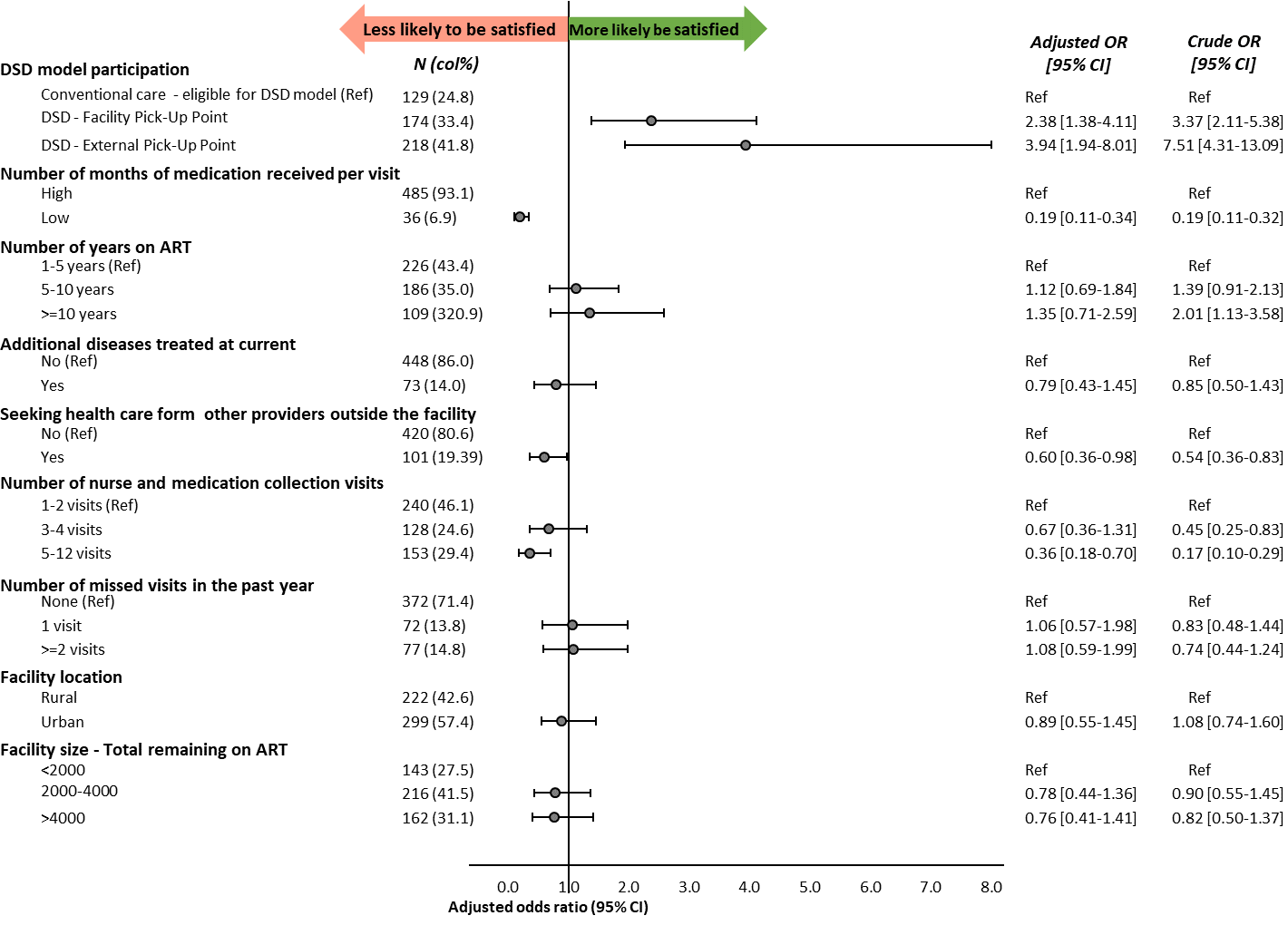


*Adjusted by perceived quality of care, time on ART, seeking outside healthcare, additional diseases treated at the facility, annual number of clinic visits, annual number of missed visits, facility location and facility size.
