# Supplementary material for: Satisfaction with service delivery among HIV treatment clients enrolled in differentiated and conventional models of care in South Africa: a baseline survey
Source: J Int AIDS Soc. 2024 Mar 25;27(3):e26233. doi: 10.1002/jia2.26233 (PMC10963588; doi:10.1002/jia2.26233)
Supplement: Supplementary file 5 — Figure S4. Gender stratified analysis [file JIA2-27-e26233-s001.docx]

Figure S4. Crude and adjusted* odds ratios of client satisfaction for DSD model and conventional care eligible study participants stratified by gender.

**Female participant (n=609)**


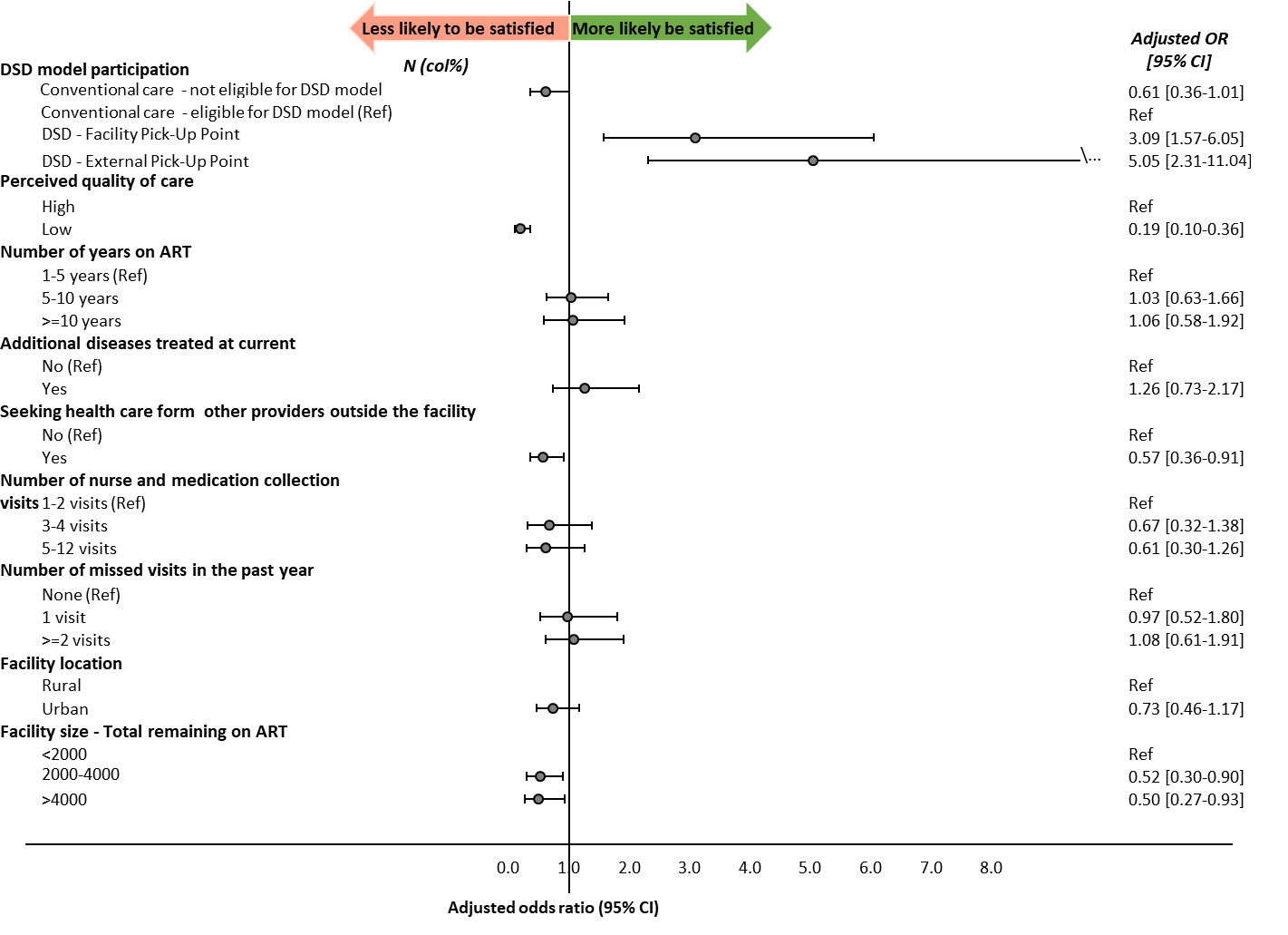

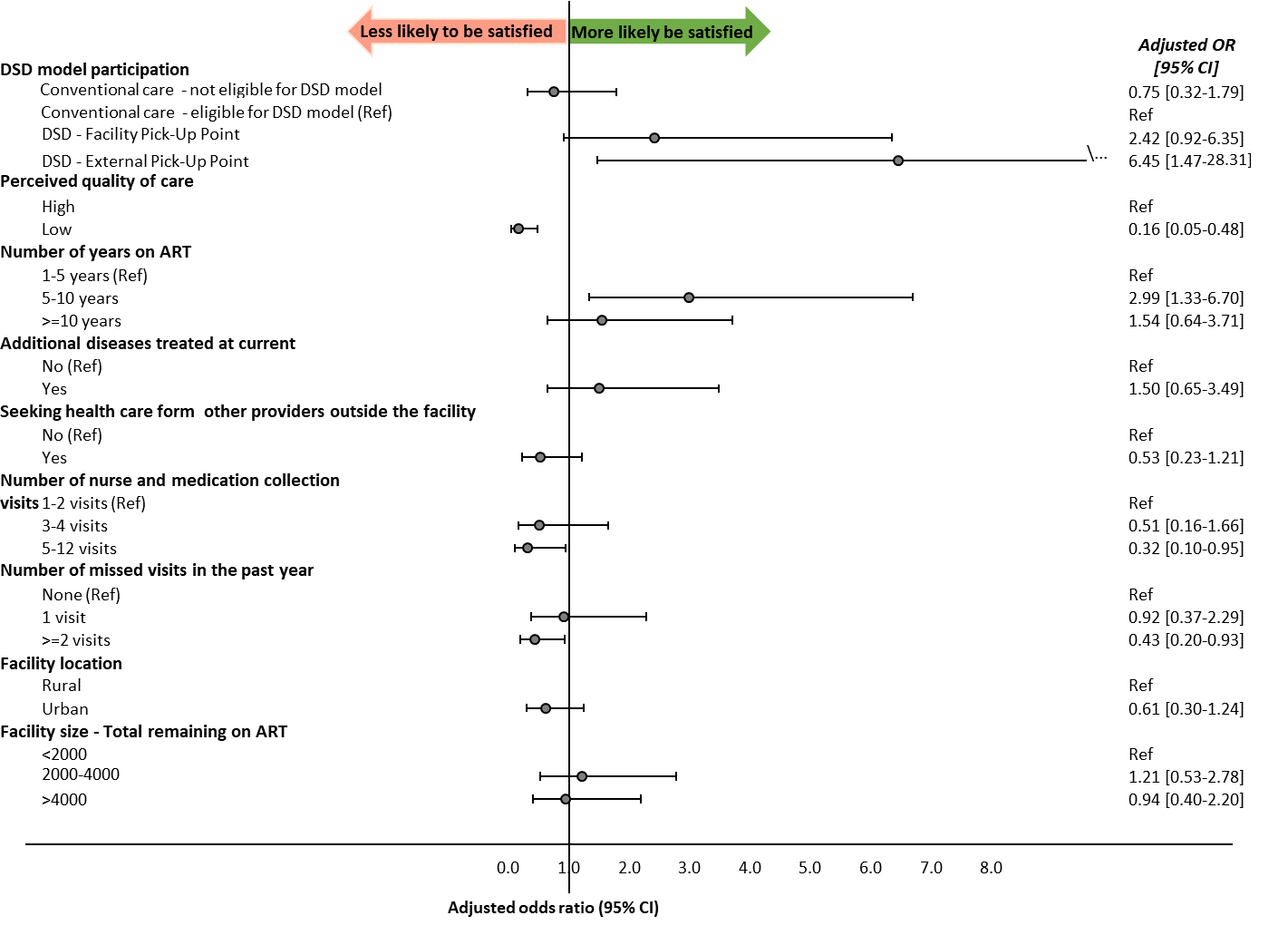


**Male participants (n=258)**

*Adjusted by perceived quality of care, time on ART, seeking outside healthcare, additional diseases treated at the facility, annual number of clinic visits, annual number of missed visits, facility location and facility size.
