# Supplementary material for: Satisfaction with service delivery among HIV treatment clients enrolled in differentiated and conventional models of care in South Africa: a baseline survey
Source: J Int AIDS Soc. 2024 Mar 25;27(3):e26233. doi: 10.1002/jia2.26233 (PMC10963588; doi:10.1002/jia2.26233)
Supplement: Supplementary file 6 — Table S1. Characteristics of the SENTINEL study sites [file JIA2-27-e26233-s005.docx]

# **Supplementary Table 1. Characteristics of the SENTINEL study sites**

| **Site** | **Setting** | | **Number on ART 2021†** | | **% of ART patients enrolled in DSD models (2021)**** |
| --- | --- | --- | --- | --- | --- |
| **South Africa** |  | |  | |  |
| *Ekurhuleni District* | |  | |  | |
| Clinic | Urban | | 2,386 | | 53% |
| Clinic | Urban | | 2,658 | | 44% |
| Clinic | Urban | | 7,213 | | 51% |
| *West Rand District** | |  | |  | |
| Clinic | Urban | | 1,783 | | Missing |
| Clinic | Rural | | 1,803 | | 32% |
| Clinic | Urban | | 1,897 | | 24% |
| Clinic | Urban | | 2,116 | | 57% |
| Clinic | Rural | | 2,301 | | 43% |
| Clinic | Urban | | 2,959 | | 66% |
| *Ehlanzeni District* |  | |  | |  |
| Community Health Centre | Urban | | 6,622 | | 61% |
| Clinic | Rural | | 3,553 | | 44% |
| Clinic | Rural | | 1,943 | | 48% |
| Clinic | Rural | | 3,001 | | 11% |
| Community Health Centre | Urban | | 5,234 | | 25% |
| Clinic | Urban | | 5,515 | | 28% |
| *King Cetshwayo District* |  | |  | |  |
| Clinic | Rural | | 1,182 | | 24% |
| Clinic | Rural | | 1,509 | | 81% |
| Clinic | Rural | | 2,231 | | 78% |
| Clinic | Rural | | 3,361 | | missing |
| Clinic | Rural | | 5,190 | | 61% |
| Clinic | Urban | | 7,934 | | 75% |

*SENTINEL clinics in the West Rand District did not have nongovernmental partners supported by PEPFAR. Sentinel clinics in the other districts received PEPFAR support from the Aurum Institute, Broadreach Healthcare, or Right to Care.

†Enrollment in DSD models as estimated by clinic staff in 2021.

**Proportion shown is for all ART patients served by the facility, including those not eligible for DSD models.
